# Study on heat transfer performance of cold plate with grid channel

**DOI:** 10.1038/s41598-024-52238-6

**Published:** 2024-02-28

**Authors:** Kaihua Zhang, Xiaojiang Ye, Zhijian Hou, Yahui Ren, Qiuyi Shi

**Affiliations:** 1 School of Mechanical and Electrical Engineering, Shenzhen Polytechnic University, Shenzhen, Guangdong China; 2 School of Optical Information and Energy Engineering, Wuhan Institute of Technology, Wuhan, Hubei China; 3 School of Energy and Environment, City University of Hong Kong, Hong Kong, China

**Keywords:** Energy science and technology, Thermoelectric devices and materials

## Abstract

The utilization of cold plate radiators as a prevalent method for indirect liquid cooling has been extensively investigated and implemented in server cooling systems. However, there is a lack of comprehensive study on the application of this technology at the chip size, indicating a need for more development and exploration in this area. A proposal was made for a grid-channel chip cold plate heat sink to facilitate the dissipation of heat from a chip. tests were conducted to investigate the impact of the flow rate of the cold plate and the layout of the inlet and outlet on various thermal parameters, including the average temperature, maximum temperature, thermal resistance, and uniformity coefficient of the cold plate. The tests were specifically conducted under a chip power of 150W, and the accuracy of the simulation was confirmed through the use of FLUENT. The findings indicate that the cold plate effectively regulates the temperature of the chip, ensuring it remains below 85 °C throughout all experimental groups. In contrast to the single in single out configuration, the single-in multiple-out layout exhibits a higher degree of temperature uniformity within the cold plate. Nevertheless, it is important to note that augmenting the quantity of exits does not guarantee an improvement in heat transfer efficiency. This outcome is contingent upon the presence of a longitudinal flow channel shared by the outlet and intake, as well as the dispersion characteristics of the outlet. Enhancing the dispersion of the exit can significantly enhance the thermal transfer efficiency of the cold plate. Furthermore, a strategy for adjusting the aperture of the orifice is proposed as a solution to address the challenges related to flow uniformity and the issue of high pressure drop in the cold plate.

## Introduction

The introduction of domestically developed chips from China has resulted in a notable enhancement in the performance of these chips. Moreover, the heat flux of these chips has experienced an increase as a result of improvements in technology. The issue of overheating presents a significant challenge in contemporary electronic equipment, namely in the domains of high-performance computing, artificial intelligence, and mobile electronics. The issue of chip overheating has significant implications for the stability and integrity of electronic components and materials, as well as the potential for component failure or damage^1,2^. As a result, the occurrence of server failure and subsequent loss of data might have detrimental effects on system security^3,4^. Consequently, the use of efficient temperature management and heat dissipation techniques are imperative in order to mitigate overheating and improve the operational stability and dependability of the microchip^5^.

The primary approaches for heat dissipation in servers are air cooling and liquid cooling^6^. Air cooling, while having a simple structure, has limited heat dissipation capacity^7^. In contrast, liquid cooling employs liquid media and radiators as a means to transport heat to the external environment, resulting in enhanced heat dissipation performance and improved energy efficiency^8^. Indirect liquid cooling technology, namely cold plate technology, has garnered considerable interest within the realm of liquid cooling technology owing to its straightforward implementation and dependable system performance. The configuration of channels within the cold plate is a crucial factor in determining the effectiveness of heat transfer. The analysis conducted by Huo et al.^9^ investigated the effects of channel number, flow direction, flow rate, and ambient temperature on a mini-channel cold plate with a parallel flow pattern. In a comparable manner, an analogous investigation was conducted in Reference^10^ to optimize the effectiveness of heat transfer. The primary differentiation lies in the convergence of the inlet and outlet inside the parallel channels.

The cold plate is a vital component in the field of indirect liquid cooling heat transfer technology, and has attracted considerable attention^11–13^. Previous research has explored the heat dissipation of the cold plate, with a focus on thermal economic analysis^14^, energy saving potential^15^, and especially structural optimization^12^. Porter Harris et al.^16^ has developed a series of spoiler structures for cold plates used in lithium battery thermal management. These structures effectively regulate the battery's temperature to ensure it remains within the optimal range throughout operation. Alfaryjat et al.^17^ conducted a comparative experiment on flow channels with varying cross sections as part of their investigation into the traditional study of flow channel topology. It was discovered that at a Reynolds number of 2200, the convective heat transfer coefficient of the circular channel may reach 1500 ${{\text{W}}/{\text{cm}}}^{2}$, while the maximum temperature of the heat source is below 75 °C. However, as compared to other experimental groups, the circular channel exhibits the most pressure drop. Song et al.^18^ examined the flow channels consisting of six trapezoidal sections. By altering the aspect ratio and flow direction of the channel section, it is determined that the heat transfer efficiency is higher in the countercurrent flow direction compared to the downstream flow direction due to the presence of greater disturbance. Raj et al.^19^ modified the topology of the flow channel to incorporate more intricate stepped and parabolic shapes. The two new types of flow channels, with their increased number of cross section angles, facilitate the formation of a gasification core compared to the traditional rectangular flow channel. This poses a challenge for the rectangular flow channel to promptly release the boiling gasification fluid under specific superheat conditions. The heat transfer rates of parabolic and stepped flow channels exhibit an increase of 88% and 169% respectively, compared to standard rectangular flow channels. Pulugundla et al.^20^ employed the time-accurate computational fluid dynamics (CFD) technique to conduct a numerical simulation of the thermal management system (TMS) for lithium batteries. A comparative analysis is conducted on the heat transfer properties of 8 distinct flow channels. Ultimately, the thermal conductivity of different cold plates is comparable, but the pressure loss caused by fluid flow varies significantly. Hence, it is imperative to take into account the decrease in pressure during the fluid flow process while constructing the structure of the flow channel. To address the issue of pressure drop in the cold plate. Wang et al.^21^ introduced many transverse gaps in the fins. These gaps serve to connect neighboring channels and improve the fluid mixing between them. These investigations have demonstrated that the cold plate has promising heat exchange capabilities and offers advantages compared to air cooled heat exchange systems.

Currently, the variety of flow channel types in cold plates is limited, with a lack of innovative structures in cold plate channels. There is a very limited number of studies examining the impact of inlet/outlet configurations on the heat transfer performance of cold plates. Research is also lacking in the characteristics and spatial arrangement of the number of outlets in cold plates. Additionally, there are few studies on methods to optimize the uniformity of fluid flow within cold plates.

This study investigates the heat dissipation performance of a grid-type-channel cold plate for a chip-scale heat source. It evaluates the influence of different inlet/outlet configurations on the cold plate's heat transfer efficiency and suggests an flow channel optimal design.

## Methods

### Experimental setup

#### Cold plate construction

**Figure 1:**
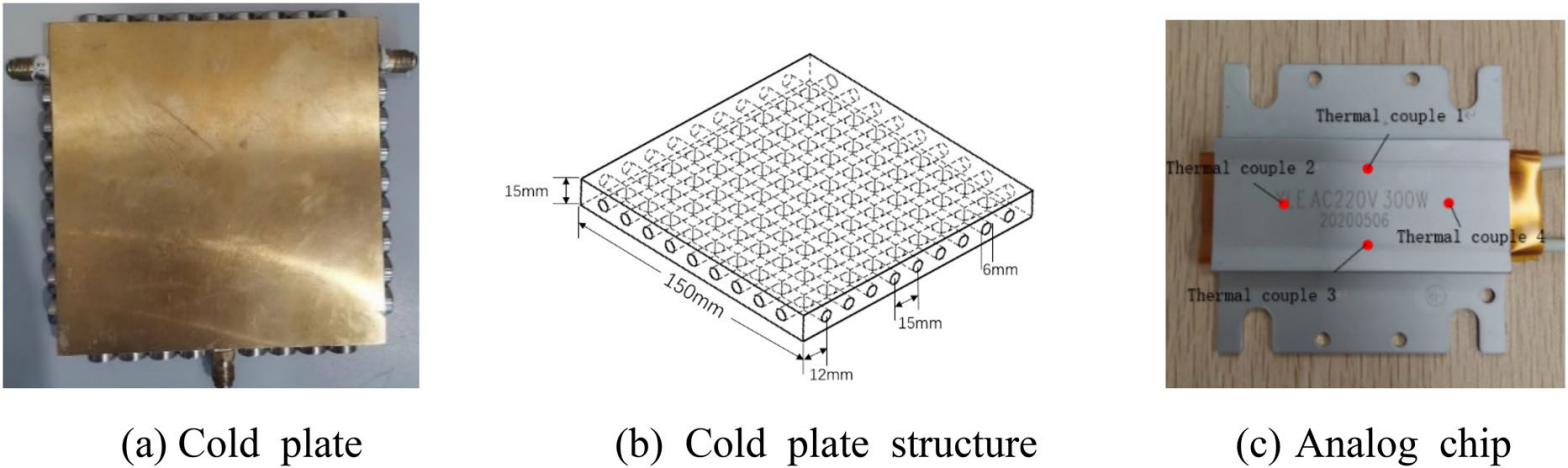
Structure of cold plate and analog chip.

Figure 1 illustrates the physical diagram of the cold plate, along with the structural and physical diagrams of the analogue chip. The cold plate under consideration is a cube made of copper, with dimensions of 150 mm × 150 mm × 15 mm. The cold plate is encompassed by nine equidistant through holes.

#### Experimental installation

Based on the trial group’s findings, the through holes of the cold plate have been effectively sealed, and the screw holes have been appropriately positioned according to the specified criteria. Additionally, the inlet and outlet of the cold plate have been connected to the circulating pump using rubber pipes. The analogue chip situated at the center of the cold plate will undergo repair using thermal grease (HY500). The thermocouple (GG-K-30) should be positioned according to the configuration depicted in Fig. 1c. Proceed by activating the power supply, adjusting the regulator to manipulate the pump and heating power of the analogue chip, and initiating the experimental procedure.

#### Experimental procedure

**Figure 2:**
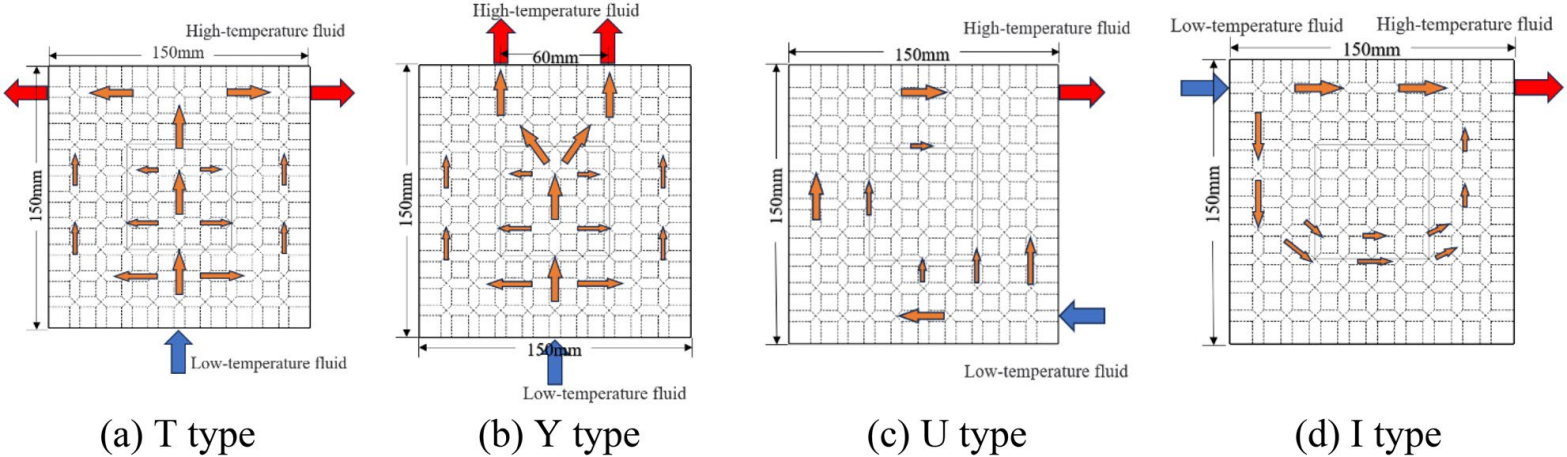
One inlet and one outlet Settings (**a**) Type T, (**b**) Type Y, (**c**) Type U, (**d**) Type I.

The ambient temperature of the cold plate is set to 25 °C. The pump is activated to facilitate water circulation within the cold plate. The flow rate is regulated by adjusting the voltage on the pump using a regulator. The heating chip is employed for the purpose of emulating heat generation, while the regulator is calibrated to maintain a constant power reading of 150W on the heating chip power. The inlet and outlet arrangement of the cold plate should be modified to Y, U, type I, as illustrated in Fig. 2. The experiment must be repeated by adjusting the distance between the two outlets of the Y-arrangement, ranging from 30 to 120 mm.

### Simulation model setup

This section employs SolidWorks for modeling research and provides a description of the cold plate model, governing equations, and boundary conditions.

#### Cold plate model

**Figure 3:**
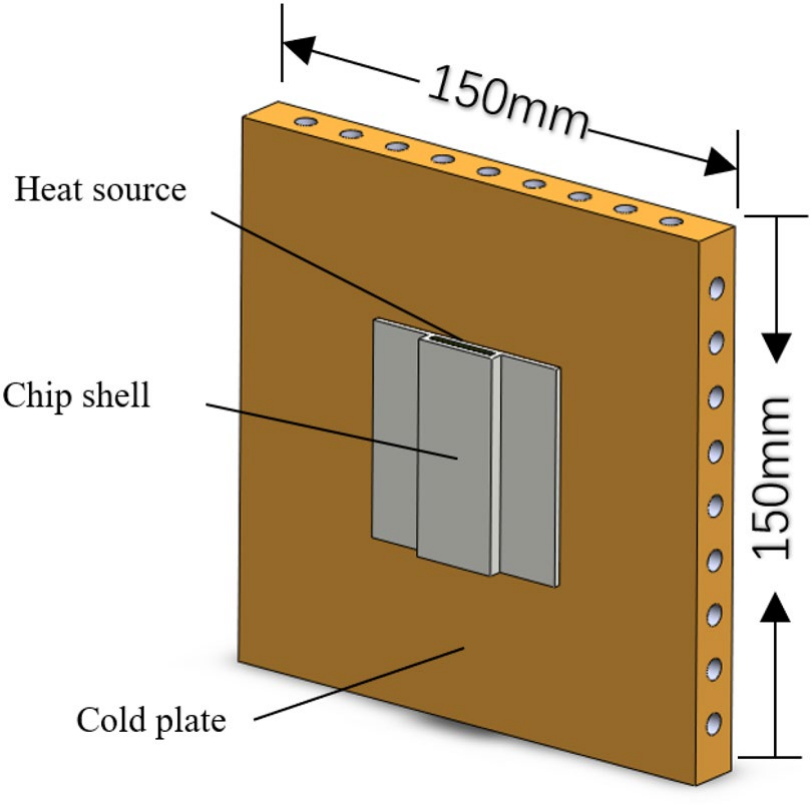
Schematic diagram of the physical model.

The schematic representation of the cold plate radiator simulation model is illustrates in Fig. 3. The entire cold plate model is a cubic structure measuring 150 mm × 150 mm × 15 mm. The model consists of four sides, each of which is perforated with a total of nine holes. These holes are evenly spaced at a distance of 15 mm from each other and have a diameter of 6 mm. The chip is located at the central region of the cold plate.

#### Flow model

For the numerical computations the below assumptions are given here: (1) the fluid(water) is considered to be uncompressible; (2) the flow process is steady-state, constant; (3) disregard the transfer of heat through convection between the system and its surroundings. (4) In the proposed model, the heat source distributes thermal energy homogeneously across the system, and the rate of heat supply remains invariant throughout the process.

The governing differential equation of the model is shown as follows:

Continuity Equation:

1 $$\frac{\partial \rho }{\partial t}+\nabla\cdot \left(\rho V\right)=0$$

momentum equation:

2 $$\rho \left(\frac{\partial V}{\partial t}+\left(V\cdot \nabla\right){\text{V}}\right)=-\nabla{\text{P}}+\rho g+\mu {\nabla}^{2}V$$

energy equation:

3 $$\frac{\partial (\rho {C}_{P}T)}{\partial x}+\nabla\cdot \left(\rho {C}_{P}TV\right)=\nabla\cdot \left({\text{k}}\nabla\cdot {\text{T}}\right)+{\text{S}}$$

where $V$ is the volume; $\rho$ is the density; ▽ Divergence; μ is viscosity; P is pressure; ${C}_{P}$ is the specific heat capacity at constant pressure; T is the temperature; k is thermal conductivity; S is the source entry.

In the present numerical study, the ANSYS CFX software (The copyrights for all commercial software applications employed in this manuscript have been duly acquired.) is used to model the turbulent flow and conjugate heat transfer. The Reynolds number (Re) from the experimental study is used to determine the nature of the flow (laminar or turbulent) based on Eq. (4).

4 $$Re= \frac{\rho vD}{\mu }$$

where $\rho$ denotes the fluid density, $v$ denotes the mean fluid velocity, $D$ denotes the hydraulic diameter and $\mu$ denotes the dynamic fluid viscosity. The Re of inlet^18^ is ${4.18\times 10}^{4}$. Therefore, turbulent flow is used in this study. The $k-\varepsilon$ turbulence model is used.

The turbulent kinetic energy and eddy viscosity equations are provided as follows:

5 $$\frac{\partial \rho k}{\partial t}=\nabla \cdot \left(\rho Vk\right)=\nabla \cdot \left[\left(\mu +\frac{{\mu }_{t}}{{\sigma }_{k}}\right)\nabla {\text{k}}\right]+{G}_{k}+{G}_{buo}-\rho \varepsilon -{Y}_{M}+{S}_{M}$$

6 $$\frac{\partial \rho \varepsilon }{\partial t}=\nabla \cdot \left(\rho V\varepsilon \right)=\nabla \cdot \left[\left(\mu +\frac{{\mu }_{t}}{{\varepsilon }_{k}}\right)\nabla \varepsilon \right]+{C}_{1}\frac{\varepsilon }{k}\left({G}_{k}+{C}_{3}{G}_{b}\right)-{C}_{2}\rho \frac{{\varepsilon }^{2}}{k}+{S}_{\varepsilon }$$

where ${C}_{1}{,C}_{2},{C}_{3}$ denote the model constants, ${S}_{M}, {S}_{\varepsilon }$ denote the UDF source terms, ${Y}_{M}$ denotes the contribution of the fluctuating dilatation in the compressible turbulence to the overall dissipation rate, ${G}_{b}$ denotes the generation of turbulence kinetic energy due to buoyancy, ${G}_{k}$ denotes the generation of turbulence kinetic energy due to the mean velocity gradients, and ${\sigma }_{k}$ and ${\varepsilon }_{k}$ denote the turbulent Prandtl numbers for $k$ and $\varepsilon$ respectively.

The turbulent viscosity is calculated as follows:

7 $${\mu }_{t}={C}_{\mu }\rho \frac{{k}^{2}}{\varepsilon }$$

Material and boundary conditions are set as shown in Table 1.

**Table 1:** Setting of boundary conditions.

Materials (cold plate)	Copper
Materials (Heat source shell)	Aluminum
Materials (heat source)	Silicon
Heat source power	150 W
External temperature	25 °C
Flowing fluid	Water
Inlet temperature	30 °C
Convection with the environment	Neglect

The convergence criteria of temperature, velocity, pressure and turbulent valuables are set as ${10}^{-3}$ in the numerical simulation.

**Figure 4:**
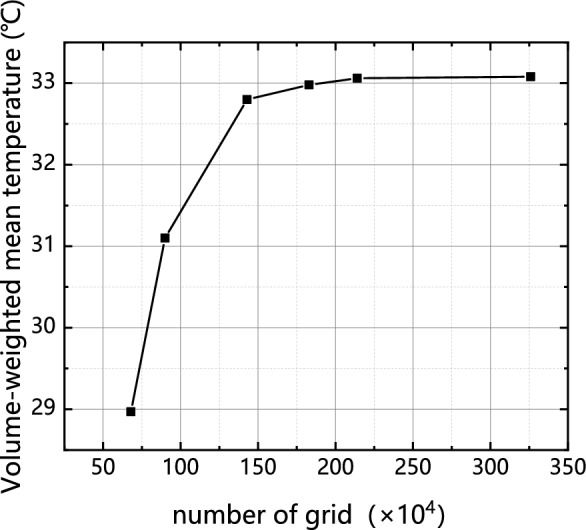
Grid independence verification result.

To ensure both calculation accuracy and efficiency, the appropriate number of mesh elements is determined, and a local mesh encryption is applied to the fluid region. For the verification of grid independence, the cold plate model in Fig. 3 was selected, and the fluid region was individually coded with a minimum mesh size of 2.97 × ${10}^{-12}{{\text{m}}}^{3}$.The present study aimed to evaluate the impact of varying mesh numbers on the computed results of the volume-weighted average temperature of the fluid within the cold plate. This investigation was conducted while maintaining a constant inlet flow rate of 43 ml/s. It is observed that thermal equilibrium is achieved when the number of grid nodes reaches 2,145,400 as shown in Fig. 4.

## Result

### Simulation validity verification

**Figure 5:**
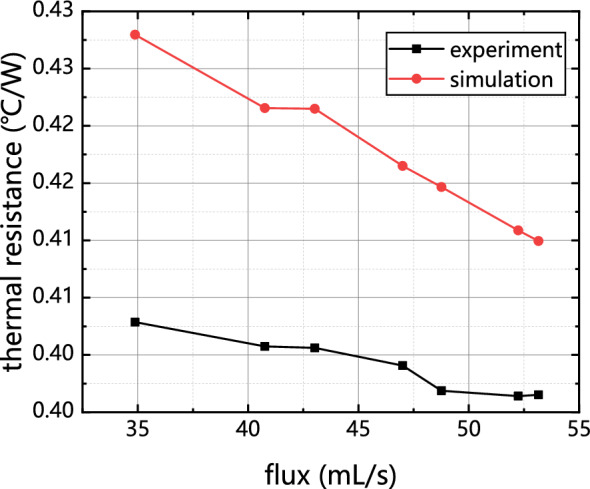
Comparison of experimental and simulated thermal resistance with flow rate.

The pressure–velocity coupling in numerical solution processes is realized by the pressure correction method based on the Coupled algorithm. In the spatial discretization of computational domains, the pressure term is discretized using the PRESTO algorithm. Figure 5 shows the trend of thermal resistance variation with flow rate in experimental and simulation test results. Thermal resistance can be defined as the resistance of the heat source in the system to transfer heat to the circulating liquid. The expression is shown as follows:

8 $$R=\frac{{T}_{source}-{T}_{f,in}}{Q}$$

where ${T}_{source}$ is the average temperature of the heat source, °C, ${T}_{f,in}$ is the liquid temperature at the inlet of the cold plate, °C, Q is heat source power, W.

The trend observed in the results indicates a consistent relationship between the simulation and experimental outcomes. Specifically, the simulation calculations neglect the convection heat transfer between the cold plate and the external environment, leading to a higher surface temperature of the chip compared to the experimental group. Consequently, this discrepancy results in an overall increase in thermal resistance. The maximum difference observed between the two approaches is only 7.65%. Therefore, the simulation is capable of providing a realistic depiction of the flow and heat transfer properties in this particular scenario.

### Influence of flow rate in T-configuration

In this paper, the heat transfer performance of the cold plate with different flow rates is compared by simulation.

**Figure 6:**
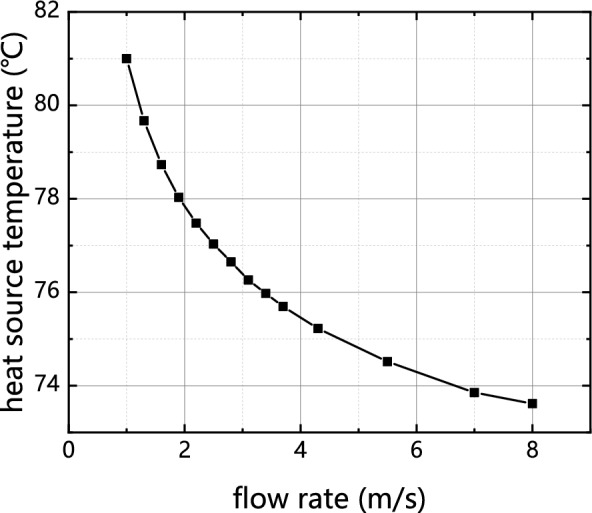
Variation of temperature of heat source with flow rate.

To ensure optimal operating performance, the maximum temperature of the chip is maintained below 85 °C. It is because that the higher temperatures have been observed to negatively impact its functionality^4^. Figure 6 illustrates the relationship between the temperature of the chip and the fluid flow within the cold plate. The results indicate that as the flow rate increases, the temperature of the chip decreases. Specifically, when the flow rate increased from 1 to 2 m/s, the chip temperature exhibited a decrease by 3.89%. However, when the flow rate further increased to 7 m/s, the chip temperature only decreased by 0.61%. Based on these findings, a flow rate of 7 m/s is selected for experimental purposes in this study.

The findings from the experiment align closely with the simulated results reported in previous scholarly works^22,23^, which investigate the impact of chip temperature on the flow rate of cold plates. The reason for this phenomenon is attributed to the significant flow resistance within the grid channel, which results in an increase in flow rate. Consequently, the pressure drop within the cold plate becomes excessively high, hindering the circulation of the fluid.

### Impact of inlet/outlet setting

#### Four arrangements

A comparison between SIMO (T and Y) and SISO (U and I) was made for the proposed grid-type-channel cold plate, and the liquid flow in the cold plate was studied by FLUENT software.

**Figure 7:**
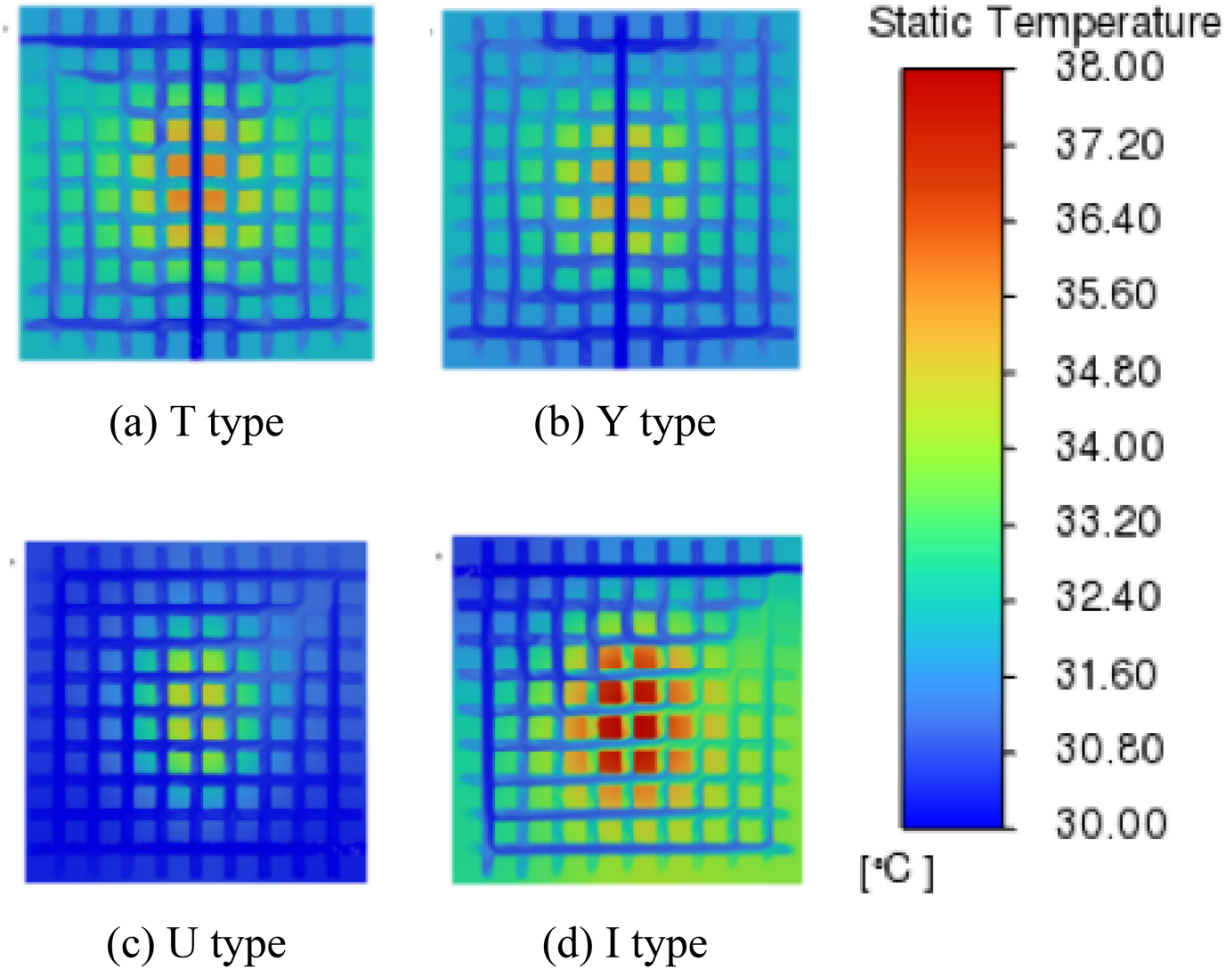
Temperature cloud image of cold plate section.

**Figure 8:**
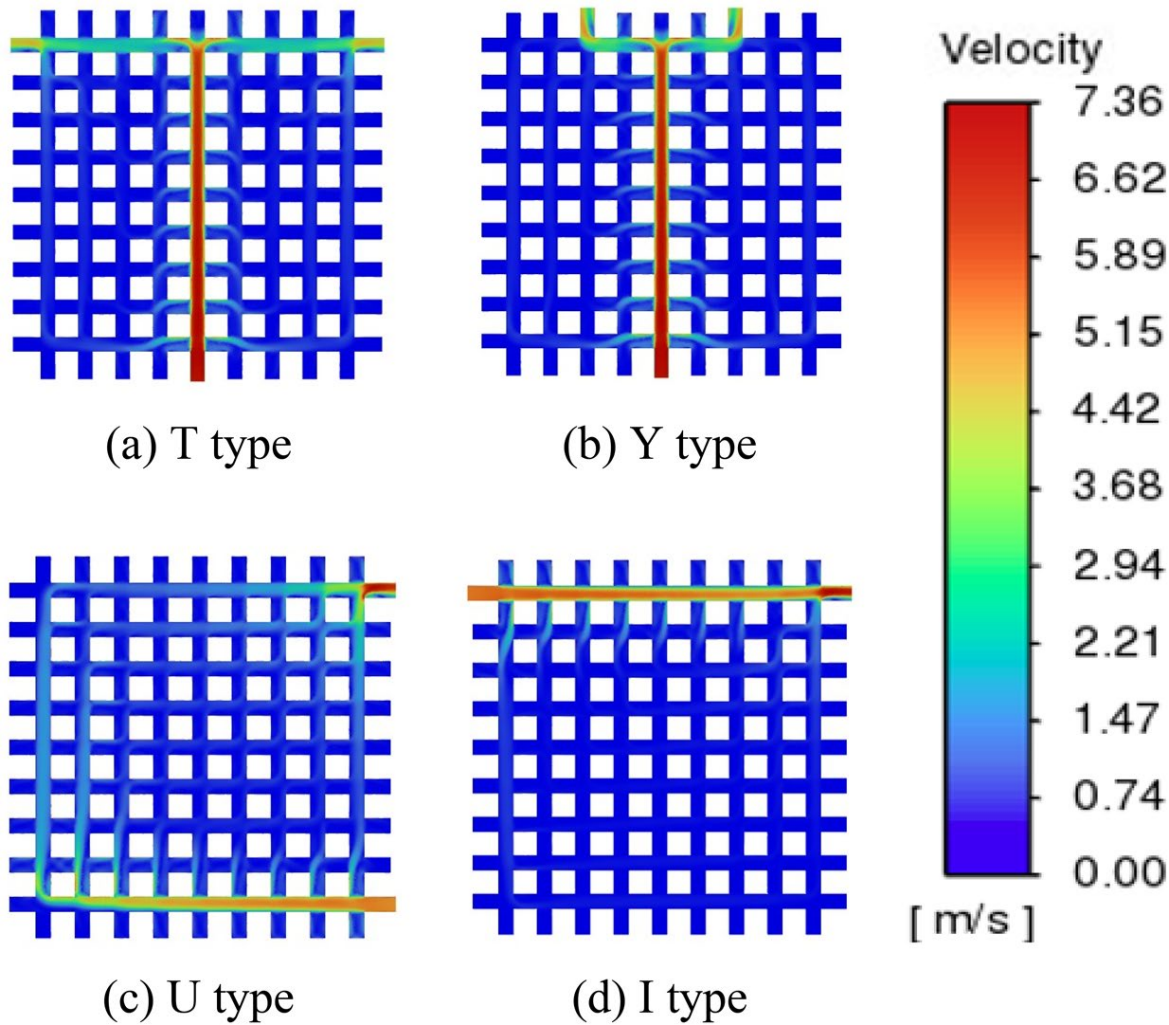
Velocity cloud image of cold plate section.

Various configurations of the inlet/outlet are simulated, resulting in the generation of a temperature and velocity cloud image (Figs. 7, 8). The uniformity of water temperature in the cold plate is enhanced while employing T-shaped and Y-shaped arrangements. From the velocity cloud image, it can be seen that the T-shaped and Y-shaped inlet and arrangements can better transport fluid to the cold plate.

The mean of uniformity coefficient is one of the indexes to evaluate the uniformity of the surface temperature of an object^24^. A higher value indicates a poorer mean temperature on the covered side. The calculation method is shown as follows:

9 $$\mu =\frac{\sqrt{\frac{1}{N} \sum_{i=1}^{N}{\left({T}_{i}-{T}_{ave}\right)}^{2}}}{{T}_{ave}}$$

where N is the number of observation point; ${T}_{i}$ is the temperature of each point; ${T}_{ave}$ is the average temperature of each point.

This study assesses the four essential performance indicators (KPIs) listed in Table 2, namely chip temperature, cold plate temperature, cold plate uniformity coefficient, and thermal resistance. The objective is to analyze the impact of inlet/outlet configuration on the heat.

**Table 2:** Four kinds of KPI arrangements and their value.

Arrangement KPI	SIMO		SISO	
	T	Y	U	I
Chip temperature (°C)	73.1	73.4	74.2	76.5
Cold plate temperature (°C)	31.4	31.0	30.9	32.0
Cold plate uniformity coefficient × ${10}^{-3}$	4.64	4.45	6.97	7.88
Thermal resistance × ${10}^{-2} (^\circ {\text{C}}/{\text{W}})$	3.91	4.02	4.37	5.79

As can be seen in Table 2, SIMO system has obvious advantages in all parameters compared with SISO system, and the average temperature coefficient of cold plate has a maximum difference of 73.83%. The maximum thermal resistance difference is 48.15%.

### The influence of outlet quantity and outlet dispersion

This section will change the outlet quantity of cold plate and the setting mode of outlet. The specific setting mode is shown in Table 3 (this study only focuses on the axisymmetric outlet arrangement mode). The objective is to investigate the influence of these modifications on the heat transfer performance of cold plate. Standard deviations are introduced to assess the dispersion of outlets in various Settings. Specific methods are shown as follows.

**Table 3:** Summary of outlet quantity and setting scheme.

Number of outlet	No. of outlet					
2	1,1	2,2	3,3	4,4	–	–
3	1,0,1	2,0,2	3,0,3	4,0,4	–	–
4	4,3,3,4	3,2,2,3	2,1,1,2	4,2,2,4	3,1,1,3	4,1,1,4
5	4,3,0,3,4	3,2,0,2,3	2,1,0,1,2	4,2,0,2,4	3,1,0,1,3	4,1,0,1,4
6	4,3,2,2,3,4	3,2,1,1,2,3	4,2,1,1,2,4	4,3,1,1,3,4	–	–
7	4,3,2,0,2,3,4	3,2,1,0,1,2,3	4,2,1,0,1,2,4	4,3,1,0,1,3,4	–	–
8	4,3,2,1,1,2,3,4	–	–	–	–	–
9	4,3,2,1,0,1,2,3,4	–	–	–	–	–

**Figure 9:**
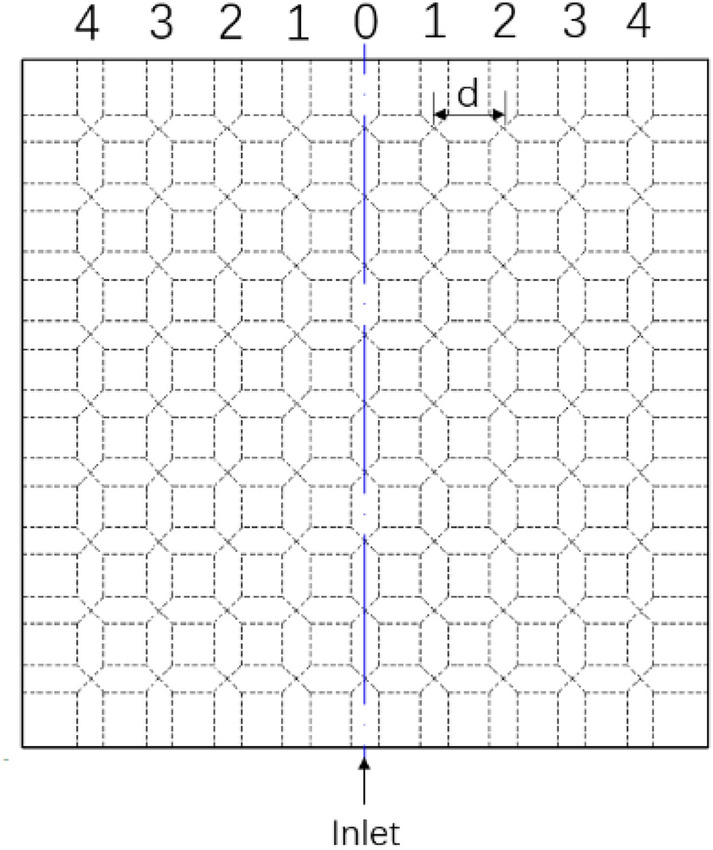
Schematic diagram of each outlet number of cold plate.

Standard deviation:

10 $$\updelta =\sqrt{\frac{{\sum }_{i=1}^{n}{ d}^{2} }{n}}$$

where d is the distance between the outlet and the symmetry axis of the cold plate which is shown in Fig. 9.

#### The impact of quantity of outlet

The influence of varying outlet quantity on heat transfer was assessed by computing the average temperature coefficient of the cold plate and the average temperature of the chip for each outlet quantity group in Table 3, using all setting techniques.

**Figure 10:**
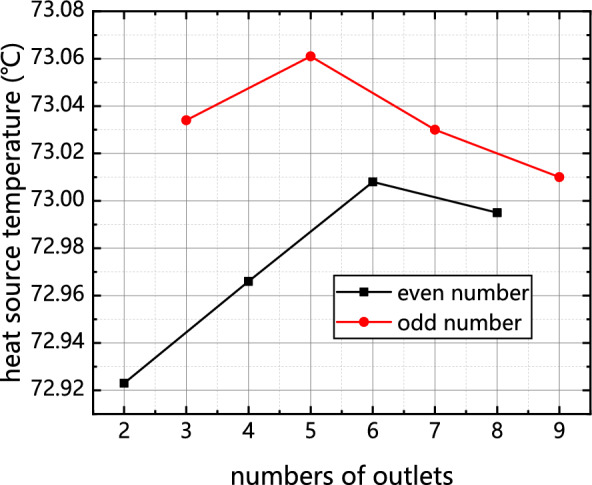
Influence of the number of outlets on chip temperature.

**Figure 11:**
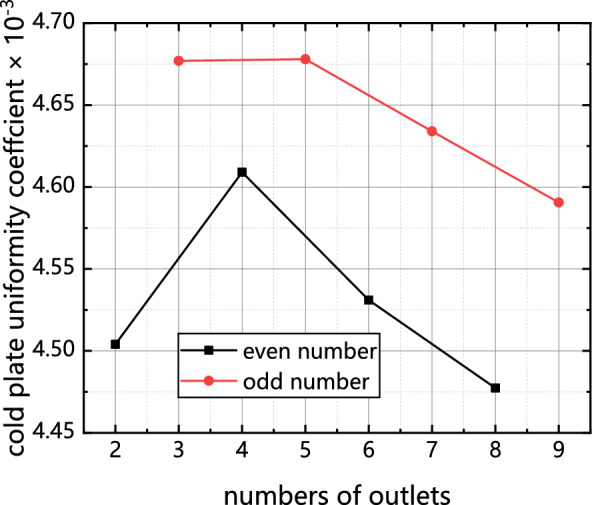
Influence of outlet quantity on uniformity coefficient of cold plate.

As illustrated in Figs. 10 and 11, there is a discernible trend wherein the average temperature coefficient of the cold plate and the temperature of the chip initially increase and thereafter decrease as the number of outlets is augmented.

Figures 10 and 11 demonstrate that the heat transfer efficiency of the cold plate with an odd number of outlets is much lower compared to the cold plate with an even number of outlets.

According to the continuity equation, if the entrance flow is determined, the outlet flow is assured. However, in this investigation, different quantities of exits will have varied configurations. These diverse layouts have a considerable influence on the flow of the fluid in the cold plate. As indicated above, since this work only covers symmetrical exit Settings, when the number of cold plate exits is odd, the cold plate exit No. 0 (Fig. 9) will certainly be opened. In Table 4, it can be shown that in all odd exit Settings, outlet 0 in all cases accounts for more than 66% of the entire Mass flow rate. The outlet and the entry share a longitudinal flow channel, resulting in a large volume of flow cannot flow through the cold plate and out of other outlets, which deteriorates the temperature equalization and heat exchange capacity of the cold plate.

**Table 4:** Flow characteristics of arrangement in odd number outlets.

	Number of outlet	No	Outlet 0 flow rate (${\times 10}^{-3}$ kg/s)	Other outlets flow rate (${\times 10}^{-3}$ kg/s)	Flow ratio of outlet 0 (%)
Mass flow rate (kg/s)	3 Outlets	4,0,4	131.81	62.52	67.9
		3,0,3	131.14	62.71	67.6
		2,0,2	131.82	62.51	67.9
		1,0,1	132.83	61.76	68.5
	5 Outlets	4,3,0,3,4	128.64	65.71	66.3
		3,2,0,2,3	128.37	65.72	66.2
		2,1,0,1,2	129.99	64.30	67.0
		4,2,0,2,4	128.90	65.48	66.4
		3,1,0,1,3	129.25	65.07	66.6
		4,1,0,1,4	129.51	64.81	66.8
	7 Outlets	3,2,1,0,1,2,3	128.60	65.19	66.3
		4,2,1,0,1,2,4	128.46	65.86	66.2
		4,3,1,0,1,3,4	128.93	65.87	66.5
		4,3,2,0,2,3,4	128.30	66.03	66.1
	9 Outlets	4,3,2,1,0,1,2,3,4	128.47	65.86	66.2

#### Impact of outlet dispersion

**Figure 12:**
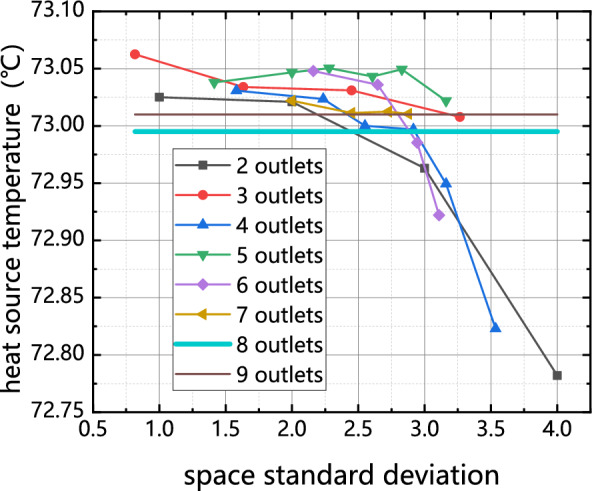
Influence of outlet dispersion degree on chip temperature.

The influence of output dispersion on chip temperature is presented in Fig. 12. The graph represents the single setting method for 8 and 9 outlets through the use of horizontal lines. It can be observed that the chip temperature remains relatively close to 73 °C for each outlet quantity. Furthermore, it is observed that the cooling effect of the cold plate positioned at 2–7 outlets on the chip diminishes to varying extents as the outlet dispersion degree increases. One notable optimization among the options is the implementation of the cold plate set designed for the 2 outlets. It is evident that this configuration, known as the I configuration, has the poorest relative heat transfer performance. However, it is capable of reducing the temperature by a maximum of 4.89%. This observation demonstrates a positive correlation between the degree of dispersion at the outlet of the cold plate and its heat transfer capability.

#### The optimization of the flow channel

**Figure 13:**
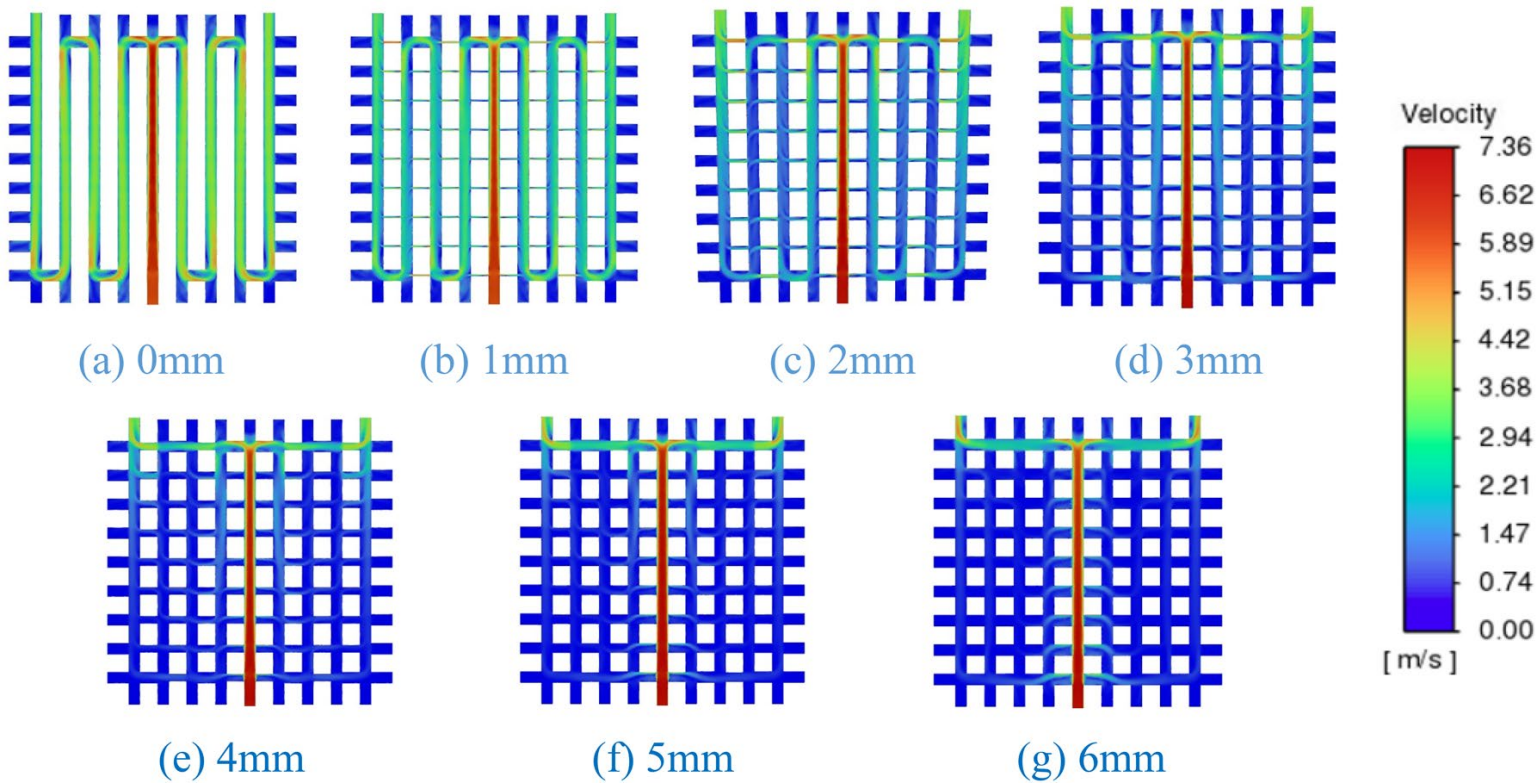
Velocity contour map for flow channel optimization.

In Fig. 7, where the T and Y configurations of the 0th flow channel (Fig. 9) exhibit minimal fluid flow on both sides. This is attributed to the uniform aperture size across the grid-style cold plate, preventing effective fluid guidance for uniform flow within the cold plate. We elected to block specific channels within the cold plate to direct the fluid in an S-shape (as shown in Fig. 13), but this resulted in a significant increase in pressure drop. According to the research by Wang et al.^21^, adding gaps to the fins helps alter the overall pressure drop of the heat exchanger. In subsequent studies, we reduced the diameter of the pipes between the S-shaped flow channels, treating them as gaps, which achieved the desired uniformity in cold plate flow. The temperature variation in each chip remained relatively minor (Table 5). It was observed that when the gap exceeded 3 mm, the effectiveness of flow guidance greatly diminished, with the lowest pressure drop reaching 9632 Pa (Table 5).

**Table 5:** Parametric comparison of flow channel optimization.

KPI	Gap size (mm)						
	0	1	2	3	4	5	6
Pressure drop (Pa)	75,152	52,679	24,796	9632	11,413	10,035	12,496
Chip temperature (°C)	72.4	72.6	72.8	72.7	72.9	73.2	72.8
Uniformity coefficient × ${10}^{-3}$	2.81	2.98	3.18	3.42	3.59	3.82	4.02

## Discussion

Based on the analysis conducted, it can be observed that the distribution of water temperature in the cold plate is more homogeneous when the arrangement is either T-shaped or Y-shaped. The preference for the SIMO configuration is attributed to its ability to facilitate a consistent liquid flow within the cold plate. The chip has a comparatively low temperature. The limited proximity of the inlet/outlet to the chip component results in a minimal direct flow of fluid through the chip. Consequently, the circulation of liquid in the U-shaped configuration of the cold plate is more comprehensive compared to the I-shaped configuration. This enhanced circulation contributes to the lower temperature of the liquid in the U-shaped cold plate. In the SISO type design, the majority of the liquid exhibits challenges in effectively engaging in the circulation process to facilitate heat dissipation. Consequently, this leads to the accumulation of heat in the bottom region of the chip, exacerbating the overall heat dissipation issue.

The disparity observed in Table 2 can be attributed to the distinct arrangement of multiple outlets in the SIMO system. This arrangement allows for the fluid to be directed to various sections of the cold plate, enabling multiple outlets to share the burden of discharging heat. In contrast, the SISO system relies on a single outlet to handle the entire load of discharged heat. Therefore, the SISO system faces challenges in effectively dissipating heat in the cold plate, leading to increased temperatures and a deterioration in heat transfer efficiency.

Based on the analysis of Figs. 10 and 11, it is observed that when the outlet reaches a height of 4–6 units, the primary flow of the mainstream fluid is concentrated near the outlet and in close proximity to the symmetry axis. This concentration of flow restricts the diffusion of heat throughout the entire cold plate. As the number of outlets increases, the nearest outlet to the symmetry axis moves further away. This means that the flow of the mainstream fluid becomes less concentrated near the symmetry axis, allowing for better distribution of heat throughout the cold plate. The increased dispersion of outlets helps to enhance the overall heat transfer capability of the cold plate. It is important to note that the inflow rate remains constant throughout this analysis, indicating that the changes in heat distribution and heat transfer efficiency are primarily influenced by the arrangement and dispersion of the outlets on the cold plate.

As previously mentioned, the diffusion of heat within the entire cold plate is impeded. As the number of outlets continues to increase, the proximity of the nearest outlet to the symmetry axis decreases. Consequently, the pressure drop experienced during outflow decreases, causing the flow near the symmetry axis to become saturated. This saturation forces the fluid to exit through outlets located at a greater distance from the symmetry axis. Furthermore, in the case of a cold plate with an odd number of outlets, the outlet that has a longitudinal flow route with the entry will be opened, resulting in a number of water was discharged from outlet 0. As a result, a significant quantity of fluid (more than 66%) was observed to outlet through outlet 0, thereby exacerbating the heat transfer efficiency of the cold plate. Additionally, it can be inferred from this observation that the presence of an odd number of outlets in a cold plate has a comparatively lesser impact on temperature uniformity and chip temperature when compared to cold plates with an even number of outlets.

To improve the uniformity of fluid flow within the grid cold plate, we reduced the diameter of certain flow channels as shown in Fig. 13, effectively utilizing these narrowed sections as gaps to guide the direction of fluid flow within the cold plate. This approach, under conditions where the temperature differential of the heat source was not significant, effectively resolved the issue of excessive pressure drop in the S-shaped flow channels. It reduced the pressure drop in the cold plate to as low as 9632 Pa and significantly enhanced the uniformity of flow and temperature distribution within the cold plate.

## Conclusion

Through the evaluation of the heat transfer performance of a grid-type-channel cold plate chip heat exchanger, the following results have been derived:

1. The grid-type-channel cold plate developed in this study underwent experimental testing and fluid simulation. The simulation results validated the accuracy of the simulation by demonstrating a maximum residual error of only 7.65%.
2. As the flow rate increases, the rate at which the chip temperature decreases is reduced. Specifically, when the flow rate is increased from 7 to 8 m/s, the chip temperature experiences a decrease of only 0.61%.
3. A comparison was made between the heat exchange performance of SIMO (Single Inlet Multiple Outlet) and SISO (Single Inlet Single Outlet) cold plates. Due to the inability of the SISO configuration to uniformly distribute high temperature fluid across the whole cold plate, the heat exchange efficiency was significantly lower compared to that of the SIMO system. Consequently, the average temperature coefficient of the cold plate exhibited a maximum deviation of 73.83%. The disparity in heat resistance reaches a maximum of 48.15%.
4. The rise in the quantity of outlets has the potential to negatively impact the heat exchange efficiency of the cold plate within the range of 4–6 outlets. Conversely, as the appropriate number of outlets continues to increase within the range of 6–9, the heat exchange performance of the cold plate is expected to improve. The heat transfer performance of the cold plate is compromised when the inlet and outlet are aligned along the same longitudinal flow path. This limitation arises from the predominant flow of fluid down this single path, preventing effective distribution across the entire cold plate.
5. The concept of outlet dispersion was introduced, and a comparison was made about the variations in chip temperature under different conditions of outlet quantities and designs. The study revealed that as the dispersion of the outlet increased, the heat emitted by the chip heat source dropped to varying extents in all cases. Among the various configurations, it was seen that the cold plate set designed for outlet 2 exhibited the most significant optimization. Furthermore, the type I arrangement, which displayed the poorest relative heat transfer performance, achieved a temperature reduction of up to 4.89%. This observation demonstrates a positive correlation between the degree of dispersion at the outlet of the cold plate and its heat transfer capability.
6. By altering the diameters of flow channels in different parts of the cold plate to guide fluid flow, and employing small-diameter gaps between these channels, we can effectively reduce the flow pressure drop in the cold plate. This approach also significantly enhances the uniformity of fluid flow and temperature distribution within the cold plate.

In summary, this study assessed the heat transfer performance of the grid cold plate and determined the impact of flow rate, arrangement mode, inlet/outlet configurations on chip heat dissipation and optimization of the flow channel. Additionally, optimal arrangement conditions were proposed to achieve optimal heat dissipation efficiency. The cold plate under consideration demonstrates efficient heat transfer capabilities to chip-scale heat sources, hence offering a promising approach for addressing semiconductor heat dissipation challenges.

## Data Availability

The datasets used and/or analysed during the current study available from the corresponding author on reasonable request.
